# The influence of the few: a stable ‘oligarchy’ controls information flow in house-hunting ants

**DOI:** 10.1098/rspb.2017.2726

**Published:** 2018-02-14

**Authors:** Thomas O. Richardson, Charles Mullon, James A. R. Marshall, Nigel R. Franks, Thomas Schlegel

**Affiliations:** 1 Department of Ecology and Evolution, University of Lausanne, Lausanne, Switzerland; 2 School of Biological Sciences, University of Bristol, Bristol, UK; 3 Department of Computer Science and Kroto Research Institute, University of Sheffield, Sheffield, UK

**Keywords:** social insect, network analysis, communication, division of labour, animal behaviour, decision-making

## Abstract

Animals that live together in groups often face difficult choices, such as which food resource to exploit, or which direction to flee in response to a predator. When there are costs associated with deadlock or group fragmentation, it is essential that the group achieves a consensus decision. Here, we study consensus formation in emigrating ant colonies faced with a binary choice between two identical nest-sites. By individually tagging each ant with a unique radio-frequency identification microchip, and then recording all ant-to-ant ‘tandem runs’—stereotyped physical interactions that communicate information about potential nest-sites—we assembled the networks that trace the spread of consensus throughout the colony. Through repeated emigrations, we show that both the order in which these networks are assembled and the position of each individual within them are consistent from emigration to emigration. We demonstrate that the formation of the consensus is delegated to an influential but exclusive minority of highly active individuals—an ‘oligarchy’—which is further divided into two subgroups, each specialized upon a different tandem running role. Finally, we show that communication primarily occurs between subgroups not within them, and further, that such between-group communication is more efficient than within-group communication.

## 1. Introduction

One of the most important life challenges faced by any animal is to find a new place to live when the current nest site becomes uninhabitable. When individuals live together in groups this challenge becomes even more demanding as, if they are to avoid group fragmentation, they must ensure that they effectively coordinate their individual actions to a single purpose.

Although group living is associated with a range of benefits, the shared occupation of a communal nesting site is also likely to induce costs associated with nest degradation, colony growth and parasitism. Consequently, group-living species often perform an emigration in which the entire society relocates to a new nest site [1]. The mechanisms that coordinate nest-site selection and colony emigration have been most thoroughly studied in ants of the genus *Temnothorax* [2–4] and in the honey bee [5,6]. In colonies of *Temnothorax* ants a key stage in colony emigration—disseminating information about potential nest-sites—is organized via stereotyped physical interactions in which a knowledgeable individual physically leads a naive nestmate back to a new nest site, in what is called a tandem run [7–10]. Such followers learn the location of the new nest site to which they were led, and later lead other ants back to the same site. Tandem running therefore serves as a vehicle for knowledgeable individuals to transmit information about suitable nest sites to nestmates.

In this paper, we present a series of experiments in which colonies of *Temnothorax albipennis* ants were repeatedly challenged with a difficult consensus-formation task—selecting between two identical nest sites. In order to trace the propagation of information about the available nest sites within the ant population, each ant was tagged with a unique radio-frequency identification (RFID) microchip and the identities of both individuals participating in each tandem run were recorded. By representing each tandem run as a directed link from the leader to the follower, we are able to reconstruct the network of social interactions underpinning the group decision.

The results are divided into three parts. In the first, we show that tandem running activity is concentrated within a minority with a consistent membership. The second section describes the basic topological features of the tandem recruitment networks formed by the minority that participates in tandem running. Finally, we examine how the role specializations of the leader and follower within each tandem pair, interact to determine the quality of the tandem run.

## 2. Methods

### (a) Experimental methods

Six colonies of *Temnothorax albipennis* ants were collected in September 2011 from the Dorset coast, UK. Colony population sizes ranged between 72 and 113 workers (mean = 88, s.d. = 13.1), and all colonies possessed brood of all stages. Prior to the experiments, all ants were individually tagged with RFID micro transponders (electronic supplementary material, figure S1; 500 × 500 × 120 μm, mean weight 89 μg; PharmaSeq, NJ, USA), according to established protocols [11]. Individuals that had groomed off the RFID tags were removed from the colony and re-tagged. To minimize any adverse effects of repeated tagging (particularly, repeated exposure to the anaesthetic, CO_2_), ants that removed their tags after the fourth tagging attempt were permanently removed from the colony (resulting in the removal of an average of 7.1% (s.d. = 4.9) of ants per colony).

The colonies were initially housed in small nests (25 × 30 × 1 mm), with a single entrance (1.2 mm wide, 10 mm long), and a transparent acetate roof allowing light into the interior nest cavity, fitted with a regular grid of holes (approximately 0.5 mm diameter, and a density of 45 holes per cm^2^). *Temnothorax* ants behave as though such nests are of poor quality [12] and when presented with better alternatives, colonies will ‘move to improve’ [13]. Colonies were presented with a choice between two ‘luxury’ nests of identical quality. These nests had twice the volume of the initial nest, (30 × 50 × 1 mm), a single-standard entrance, and were covered with cardboard ceiling so that their interiors were dark.

All emigrations were started between 10.00 and 12.00. On the morning of each emigration the initial nest was placed into a rectangular arena (45 × 75 cm), and the three nests arranged into an equilateral-triangle, with the two luxury nests—we term them ‘left’ and ‘right’—placed on either side of the original (electronic supplementary material, figure S1). During each emigration, a handheld RFID reader was used to record the identity of the nest from which each tandem departed, the associated tandem start and end times, and the identities of both participants. A high-resolution video camera placed above the arena was used to record the emigration and to determine whether each tandem run reached its target nest, or whether it broke up while en route.

At the end of each emigration the colony was removed from the arena and re-emigrated into their initial low-quality nest. The above emigration procedure was repeated five times for each colony. To minimize the effects of learning [14], 7 days elapsed between successive emigrations.

### (b) Defining tandem run quality

Tandem runs sometimes break up before reaching their destination. Nevertheless, partial tandem runs still convey information [9,15]. Therefore, we defined the quality of each tandem as the difference between the initial distance between the tandem starting point and the target nest, *d*, and the final distance from the end point of the tandem to the goal, *d*′, that is *d* − *d*′ (electronic supplementary material, figure S1). To arrive at a tandem quality metric that varies in the range 0–1, this difference was then normalized by the initial distance to the target, that is, *Q* = (*d* − *d*′)/*d* [16]. Hence successful tandems always had *Q* = 1, and unsuccessful tandems *Q* < 1.

### (c) Constructing tandem recruitment networks

The sequence of tandem runs that occurred during an emigration was represented as a static network in which individual ants were represented as nodes, and tandem runs were represented as directed links pointing from the tandem leader to the follower. Links were also weighted according to the tandem quality *Q*. Accordingly, the number of tandem runs that an ant led and followed respectively define its in- and out-degree centrality, whereas the sum of qualities of the tandem runs it led and followed define its *weighted* in- and out-degree centrality.

## 3. Results

### (a) Emigrations are organized by a minority with a stable membership

Participation in tandem running showed a clear division of labour, with a mean of only 29 ± 1.6% (MSE, *n* = 30 emigrations) of the colony participating. This value is in close agreement with a previous estimate of 35 ± 8% (*n* = 12) for *T. albipennis* [17], and 25.5 ± 4.1% (*n* = 6) for *T. curvispinosus* [18]. When the two tandem running roles were considered separately, participation became even more restricted, with a mean of 22.2 ± 1.4% (*n* = 30) of the colony engaging in following, and only 12.1 ± 0.8% (*n* = 30) engaging in leading.

**Figure 1.**
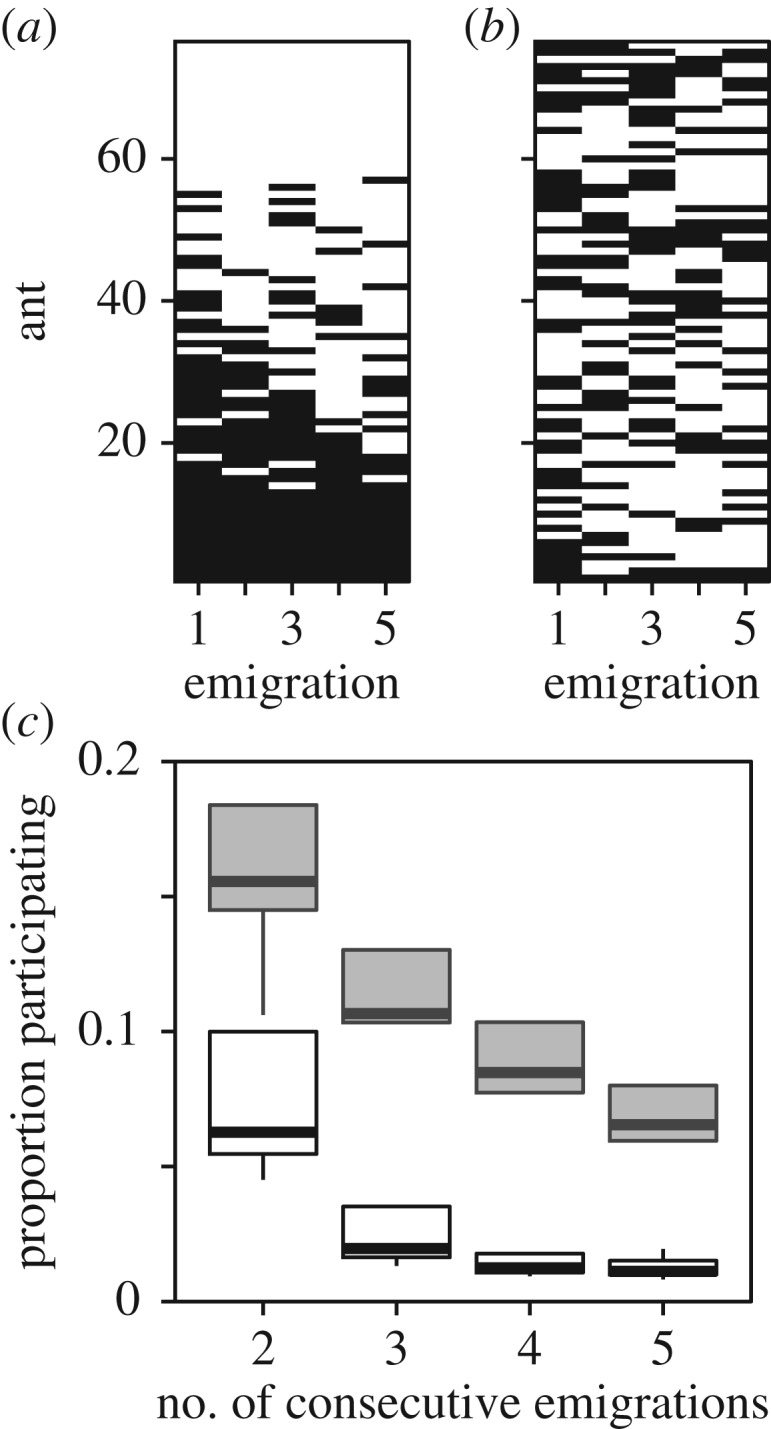
The membership of the emigration organizers is stable over multiple emigrations. (*a*) Tandem participation records for colony 1. Rows indicate the work history of a single individual over time. Black cells indicate that an individual led or followed at least one tandem run. (*b*) Synthetic version of the colony tandem participation records shown in panel (*a*), produced by randomly reshuffling the entries within each column. The degree of across-emigration consistency within the emigration organizers measured on the shuffled participation records represents the null expectation under the assumption that membership is not stable over time. (*c*) The proportion of the colony that participates in tandem running across successive emigrations (grey; observed, white; random expectation).

In order to assess the stability of the emigration organizers in *Myrmica rubra* ants, [19] performed two consecutive emigrations, and counted the number of participants common to both organizing groups. However, as the colonies here underwent five successive emigrations, in addition to measuring the proportion of the colony that participated in tandem running across successive emigration pairs (8-days between first and second emigration), we also measured participation across successive triples (16 days between first and third emigration), quartets (24 days) and quintuplets (32 days). As some individuals could have appeared in multiple emigrations just by chance, we compared the observed participation records with synthetic versions produced by randomly permuting the originals (figure 1*a*,*b*). Comparisons between the original and the synthetic participation records revealed that the observed activity records had significantly more ants common from one emigration to another than the permuted versions (figure 1*c*). Hence, the emigration organizers are a stable minority with a core membership that reliably re-assembles across multiple emigrations, that is, an ‘oligarchy’ [20].

### (b) Structure of tandem recruitment networks

**Figure 2.**
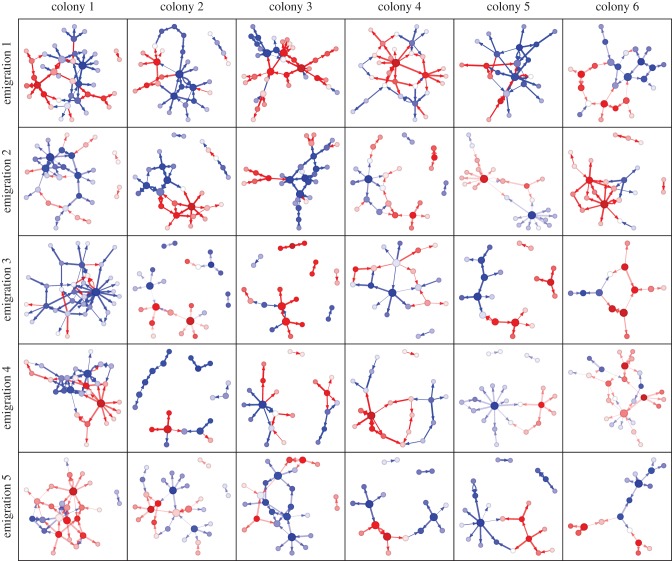
Tandem recruitment networks. Nodes represent ants and links represent tandem runs. Edge widths and shading are proportional to the quality of the tandem run, *Q*. Edge colours represent the nest target (red, left nest; blue, right). Node colours represent the weighted commitment (red, committed to the left nest; blue, right; white, neither). Node size is proportional to the weighted degree centrality, that is, the sum of all in and out edges arriving and departing from a node.

**Figure 3.**
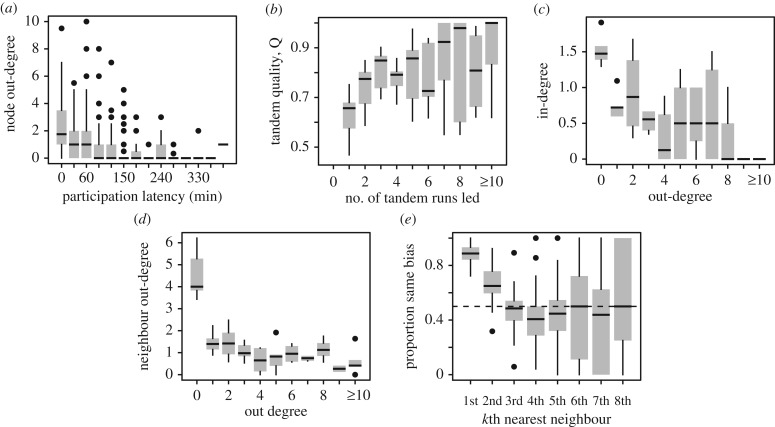
Topology of tandem recruitment networks. (*a*) Rapid reacting ants achieved high in- and out-degree centrality. (*b*) Ants became better leaders with practice. (*c*) The more tandems an ant led, the fewer it followed. (*d*) The neighbours of highly active tandem leaders were peripheral ants that led few tandems. (*e*) Local domains of bias surround each node; neighbours up to two steps away from a given node tend to share the same nest commitment.

Representing the sequence of tandem runs as a time-aggregated network revealed the presence of ‘hub’ nodes with high centrality (figure 2). As tandem recruitment is a self-reinforcing process in which leaders lead followers who may later become leaders, we first investigated whether the degree centrality of each ant was associated with its latency to begin participating in tandem running in the same emigration. We found that ant participation latency strongly predicted both node out- and in-degree centrality, that is, the number of tandems each ant led and followed respectively (figure 3*a*; linear mixed-effects model, response; out-degree, predictor; participation latency, random effects; emigration, colony identity and ant identity; effect of latency, *β* = − 0.0078 ± 0.001, *p* < 0.00001, in degree; *β* =−0.0026 ± 0.0006, *p* < 0.0001). Similarly, participation latency also predicted the weighted out- and in-degree, that is, the summed quality of tandems led and followed (weighted out-degree; *β* = − 0.005 ± 0.0009, *p* < 0.0001, weighted in-degree; *β* = − 0.0021 ± 0.0005, *p* < 0.0001). Hence, the ‘first responders’ came to occupy significantly more central positions in the tandem recruitment networks than those that took longer to participate.

As ants that engaged in several tandem runs had the opportunity to improve their leading or following skills, we next explored the association between tandem run quality and practice. Accordingly, we tested whether the quality of a tandem run depended upon the number of times the leading ant had led so far in that emigration, and the number of times that the follower had followed. Tandem run quality showed a significant positive dependence with the number of times the leading ant had so far led (figure 3*b*; LMM, response; log_10_(*Q*), effect of number of tandems leader led; *β* = 0.013 ± 0.0035, *p* = 0.00023), but no dependence upon the number of times the follower had so far followed (*β* = 0.0040 ± 0.0082, *p* = 0.63). This shows that tandem run quality is primarily determined by the amount of practice that the leader has in the leading role, but not the practice of the follower in the following role.

As the performance of individual in a particular task is typically thought to depend upon the extent to which it is specialized in that task, we next investigated whether ants specialized upon either leading or following. Because an explicit division of labour between specialist leaders and followers has not been documented in any species of ant, we expected either (i) no dependence between the average number of tandems each ant leads and the number it follows, or (ii) a positive dependence, arising from variation in activity levels. In fact, node in- and out-degree displayed a strong negative dependence (figure 3*c*; LMM, response; in-degree, predictor; out-degree, random effects as previously; effect of out-degree, *β* = − 0.17 ± 0.016, *p* < 0.00001; the potential role of time constraints in generating this negative correlation is explored—and rejected—in the next section). Hence, we conclude that at least within single emigrations ants specialized upon a single role. Indeed, this specialization is confirmed by the conspicuous ‘hub-and-spokes’ motifs visible in most of the networks (figure 2).

Since correlations between node in- and out-degrees do not provide information about the overall connectivity of the network, we next tested for associations between the centrality of each node, and the centrality of its neighbours. Given the key role of the participation latency in determining an ants' centrality, and given that the first-responders' first few generations of followers would also have ample time to accumulate a large number of tandem runs, we expected that central nodes should neighbour other central nodes. In other words, the networks should display positive degree assortativity. However, in contrast to our expectations the tandem recruitment networks displayed strong negative assortativity for node out-degree, and a weaker negative assortativity for in-degree (figure 3*d*; LMM, response; neighbour out-degree, predictor; node out-degree, random effects as previously; effect of out-degree, *β* = − 0.63 ± 0.04, *p* < 0.00001, LMM for in-degree; *β* = − 0.31 ± 0.03, *p* < 0.00001). Identical patterns were observed when node centrality was defined by the weight of the incident edges, that is, by the summed quality of the tandem runs each ant led or followed (electronic supplementary material, figure S2). Hence, the neighbours of ants that were particularly active tandem leaders tended to be particularly inactive leaders, whereas the neighbours of particularly active followers tended to be particularly inactive followers.

As well as varying in terms of their centrality, ants also varied in the extent to which they were biased towards the left or right nest site, as indicated by the number of tandems they led or followed to either site. To assess the extent to which similarly biased individuals were clustered in the recruitment networks, we quantified bias by counting the number of tandem runs to each nest that each ant had been engaged in. Hence, an ant that participated in more tandems to the left than to the right nest was classified as left biased (ants that engaged in the same number of tandems to both nests were classified as unbiased). For each node, we then measured the proportion of its nearest neighbours that shared its nest bias, and repeated this procedure for the second, third and *n*th nearest neighbours. We found that the first and second nearest neighbours tended to share the nest bias of the focal node, but from the third nearest neighbours, the agreement converged to chance levels (figure 3*e*).

Taken together, these results show that the first responders act as ‘nuclei’ around which the network is assembled. These first responders eventually become network hubs, and although they interact relatively little with one another, they nevertheless each accumulate a relatively large number of followers that together constitute a local ‘domain’ of nestmates that share the same nest commitment.

### (c) Participation order and centrality are consistent across multiple emigrations

Models of nest-site selection in social insects have successfully simulated the formation of a consensus group decision by assuming that all colony members are essentially equivalent, and that all follow the same set of rules of thumb [12,17,18,21–24]. If so, the number of tandem runs an individual leads and follows in one emigration should be independent of the number it leads at a later date. To test this prediction, we performed a time-lagged correlation analysis to test whether the performance of an individual in one emigration (at time *t*) predicts its behaviour in a later emigration (at *t* + lag). As each colony underwent five emigrations each separated by 7 days, the pairwise correlations were measured at four different time-lags (lag = 8, 16, 24, 32 days). Given that division of labour and individual behavioural specialization are universal hallmarks of social organization within colonies of social insects [25–28], and given that *Temnothorax* ants are relatively slow to mature, we predicted that individuals would exhibit strong behavioural persistence across multiple emigrations.

**Figure 4.**
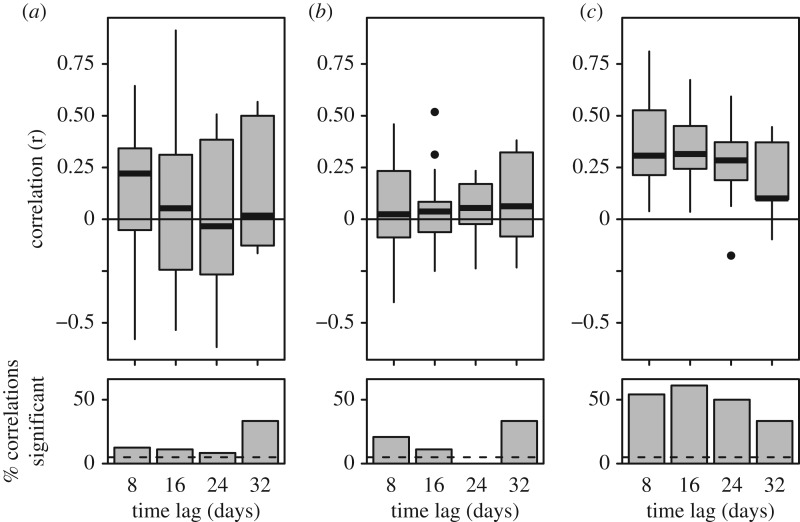
Temporal persistence of individual behaviour over repeated emigrations. (*a*) Participation latency. (*b*) In-degree. (*c*) Out-degree. Boxplots show the distribution of Pearson correlation coefficients. Each emigration combination contributes a single value to each box. Barplots show the proportion of emigration combinations that exhibited a statistically significant correlation. Dashed horizontal lines indicate the threshold for statistical significance.

Overall, the correlations were more noisy than predicted. Nevertheless, the participation latency exhibited above-chance correlations—notably so for the longest time lag (32 days; figure 4*a*). Somewhat surprisingly, in-degree correlations centred around 0 for all lags, and the statistical significance of these correlations only just exceeded chance levels for the shortest time lag (8 days; figure 4*b*). However, the out-degree correlations exhibited a clear positive bias for all time-lags (figure 4*c*). Therefore, both the order in which the recruitment network is assembled, and the ‘hubness’ of each ant within the network, are preserved from across multiple emigrations. The presence of individual consistency in the number of tandems led and followed further confirms that the negative correlation between the number of tandem runs led and followed within single emigrations (figure 3*c*) is not an artefact of constraints imposed by the finite time required to perform a tandem run.

### (d) Assortative matching within tandem pairs

In this section, we investigate an additional component of individual behavioural consistency: task reliability, which is the likelihood that an individual performs a given task when it is given an occasion to do so. The reliability for tandem leading and following (*R*_leading_, *R*_following_) was defined as the number of emigrations in which an individual led or followed at least one tandem run. Because leading and following reliability are defined in binary terms (e.g. leading or not leading), reliability reflects the extent to which a given individual can be depended upon to perform a particular task when the opportunity presents itself.

**Figure 5.**
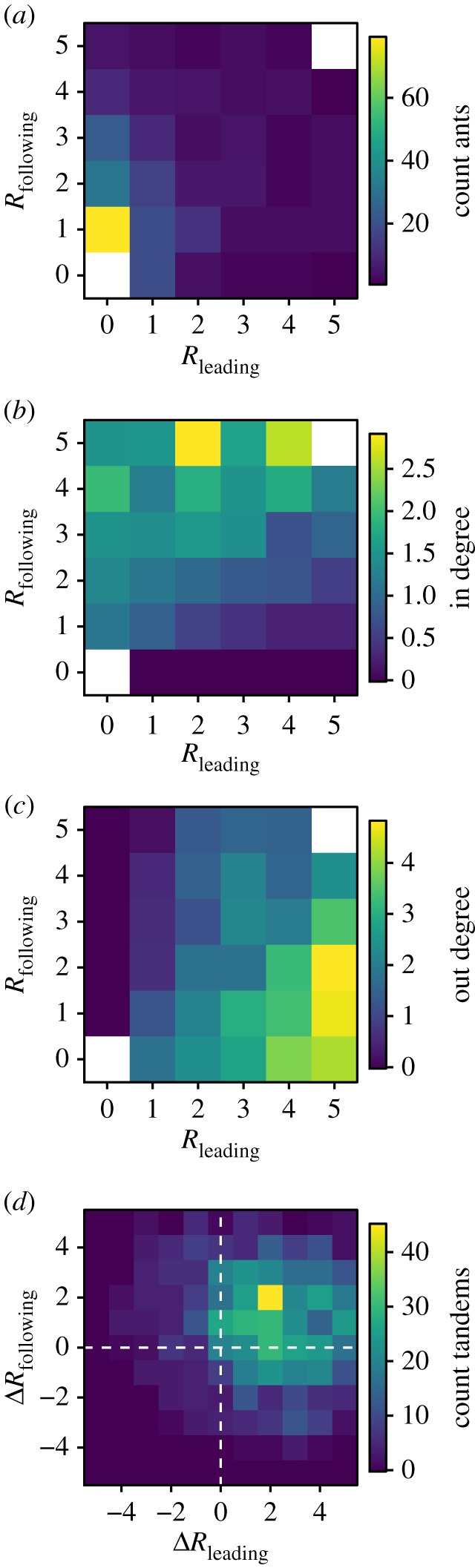
Assortative matching between tandem leaders and followers. (*a*) Joint frequency distribution of the leading and following reliability, *f*^ant^(*R*_leading_, *R*_following_). Each cell gives the count of the number of ants (over all colonies) that respectively led and followed *R*_leading_ and *R*_following_ tandems. (*b*) Ants that were reliable followers but unreliable leaders had high mean in-degree. (*c*) Ants that were reliable leaders but unreliable followers had high mean out-degree. (*d*) Joint frequency distribution of the *differences* in individual leading and following reliability between the leader and follower ant in every tandem pair, *f*^tand^_Obs_(Δ*R*_leading_, Δ*R*_following_). (Online version in colour.)

As each individual was assigned a separate reliability score for tandem leading and following, there were 36 possible combinations in total, so to examine how these scores co-varied across the worker population, we constructed the joint frequency distribution of individual role reliability, *f*^ant^(*R*_leading_, *R*_following_) (figure 5*a*). Other than individuals that led and followed in every emigration (of which there were none), the rarest category of tandem participant was that of the consistent ‘core’ oligarchy members, that is, ants that always either led or followed (*R*_leading_ = 5 or *R*_following_ = 5; 7.6% of the colony), ants that always followed but never led (*R*_leading_ = 0 & *R*_following_ = 5; 2.8%), and ants that always led but never followed (*R*_leading_ = 5 & *R*_following_ = 0; 1.9%). These reliable leaders and followers were also highly effective within their specialized role. Thus, within any given emigration, the most reliable leaders led more tandems than any other category of ant (table 1, figure 5*b*), and these tandems were of higher quality than tandems led by less reliable leaders (electronic supplementary material, figure S3), whereas the most reliable followers followed more tandems than any other category (figure 5*c*), and these tandems were of higher quality than those in which unreliable individuals followed. However, the negative association between following reliability and the quality of tandems led (table 1; electronic supplementary material, figure S3) showed that when follower specialists played the role of leader, the quality of the tandem run was lower. Therefore, while specialization within one role is associated with higher performance within that role, this may incur a cost of reduced performance in other roles. These results show that the most reliable individuals are also the most specialized, hence the core of the oligarchy is composed of individuals that each specialize upon either leading or following, but not both.

**Table 1.** Leading and following reliability predict the number and quality of tandems led and followed. Statistics report the results from linear mixed-effects models. In all models, the random effects were emigration number, colony identity and ant identity nested within colony identity. In order to achieve residual normality, the degree was square-root transformed, and the tandem quality was log_10_ transformed.

response	predictor	coefficient, *β*	s.e.	d.f.	*t*	*p*
out-degree	*R*_*L*_	0.73	0.038	760	19	<0.00001
	*R*_*F*_	−0.24	0.043	750	−5.7	<0.00001
in-degree	*R*_*L*_	−0.11	0.023	250	−4.9	<0.00001
	*R*_*F*_	0.31	0.026	290	12	<0.00001
out-quality, *Q*^out^	*R*_*L*_	0.02	0.0083	69	2.4	0.018
	*R*_*F*_	−0.02	0.0077	86	−2.6	0.011
in-quality, *Q*^in^	*R*_*L*_	−0.0039	0.006	570	−0.66	0.51
	*R*_*F*_	0.024	0.0067	570	3.7	0.00027

As the performance of tandem pair was acutely sensitive to how individuals with different levels of specialization in each role allocated themselves to leading or following duties, we next explored whether ants preferentially assorted with one another within tandem pairs according to their reliability in either role. To test for the presence of such assortative matching, we first characterized each tandem run by measuring the differences between the role reliabilities of leader and follower. Thus, for each tandem run we obtained: (i) the signed difference between the *leading* reliability of leader and follower, Δ*R*_leading_ = *R*^leader^_leading_ − *R*^follower^_leading_, which was positive if the leader of the tandem was a more reliable leader than the follower of tandem; and (ii), the signed difference between the *following* reliability of leader and follower, Δ*R*_following_ = *R*^follower^_following_ − *R*^leader^_following_, which was positive if the follower was a more reliable follower than the leader. Accordingly, each tandem run was classified according these two differences (Δ*R*_leading_, Δ*R*_following_), and the counts of the ants in each category were plotted as a joint distribution, *f*^tand^_Obs_(Δ*R*_leading_, Δ*R*_following_). Most tandem runs exhibited positive Δ*R*_leading_ and positive Δ*R*_following_ values (figure 5*d*). In other words, most tandem runs were composed of a leader that was a more reliable leader than the follower, and a follower that was a more reliable follower than the leader, which is consistent with the presence of assortative matching.

To assess whether this bias towards positive values of Δ*R*_leading_ and Δ*R*_following_ was a real phenomenon, we compared the observed distribution of leader–follower role reliability differences, with that expected in the absence of such matching (electronic supplementary material, figure S4). These comparisons revealed that the observed distribution exhibited significant bias towards the upper-right quadrant, in which leaders are reliable leaders, and followers reliable followers (goodness-of-fit test; d.f. = 114, *χ*^2^ = 2723, *p* < 0.0001). Therefore, pairs of tandem running ants are not randomly assembled, but rather their composition is consistent with a division of labour between leading and following specialists.

## 4. Discussion

Decisions made by animal groups may be placed on a continuum, extending from democratic (majority decisions) through oligarchic (minority decisions) to despotic (leader decisions) [20,29]. We have shown that group decision-making in emigrating ant colonies is controlled not by a transient assemblage that forms only for the duration of a single emigration, but rather by a stable minority association whose members each reliably play a similar role in emigration after emigration, or in other words an ‘oligarchy’. Although the exclusive control of a group's decision by one or a few older, more experienced or more dominant leaders has been documented in birds [30], wild dogs [31] and primates [32], the core ‘oligarchy’ of an emigrating ant colony is unique in that it is sub-divided into two subgroups, each specialized upon a different communication role, and further, communication within the oligarchy occurs primarily between these specialist groups rather than within them.

Our investigations into the topology of the tandem recruitment networks revealed several properties in common with networks generated by the well-known ‘preferential attachment’ model of network growth. In these models, networks are built by sequential addition of new nodes, which are more likely to be connected to the most well-connected of the existing nodes, thus creating a self-reinforcing process in which the ‘rich get richer’ [33]. Like the tandem recruitment networks studied here, networks built by preferential attachment exhibit skewed degree distributions in which there are only a few highly influential nodes and a much larger number of poorly connected nodes. Indeed, in both the tandem recruitment networks and in networks built by preferential attachment, the high-degree nodes tend to be those that were added first, whereas the lowest-degree nodes tend to be last to arrive. However, in addition to the self-reinforcing effects of preferential attachment, there are likely to be other positive feedback mechanisms acting to amplify or reinforce initial differences in centrality. For example, the positive association between the number and quality of tandem runs led demonstrates the presence of a ‘rich-get-richer’ process within single emigrations, and similarly, previous work has shown a positive association between individual experience and propensity to lead over much longer time-scales [34].

The assortative matching between experience and role that we have described in tandem running ants bears a striking resemblance to that seen in foraging stickleback fish in which the joint foraging efficiency of foraging pairs is dependent upon the difference in temperament between the leader and follower [35]. Manipulative experiments confirmed that group foraging performance was improved by increasing temperamental differences between leader and follower, but inhibited when naturally shy followers and naturally bold leaders were artificially induced to switch roles. As such, it would be most informative to use direct manipulations of tandem pair composition to establish a causal relation between group composition and performance.

Interestingly, the presence of an association between specialization and efficiency during tandem running is in contrast to the absence of such an association for foraging, brood transport and nest material collection in a closely related ant [36]. We suggest that this discrepancy derives from the additional demands placed upon individuals engaging in team tasks such as tandem running, as such tasks require several non-interchangeable individuals to do different things at the same time [37,38], while also modulating their actions according to those of the other team members. Thus, whereas a tandem follower must constantly antennate the gaster of the walking leader while simultaneously paying attention to learning the route, a tandem leader must find her way back to the target while also paying attention to the presence (or absence) of the follower. The limited cognitive abilities of the ant brain may therefore constrain the performance of generalist leaders and followers, hence it may pay for individuals to specialize upon one role or the other.

These considerations aside, the delegation of the fate of the group to an ‘oligarchy’ probably carries a degree of risk. For example, if the oligarchy members are in some sense special, then the group could be vulnerable to the loss of only one or two oligarchy members. Indeed, in the ants *Formica sanguinea*, *Camponotus sericeus* and *Diacamma indicum*, only a small minority of the workers perform the majority of the recruitment, and the removal of these recruitment specialists severely inhibits the emigration process [26,39]. Given that the entire group is vulnerable to the loss of just a few key individuals, what are the potential benefits of ‘oligarchic’ decision-making? First, the delegation of the decision to a minority could reduce time costs associated with achieving a unanimous majority decision. Second, whereas majority decision-making may reduce individual- and group level costs when there are conflicts of interest between group members [20], there is little potential for conflicts of interest during colony emigration in eusocial insects, as in such species the interests of the individual and the group are usually tightly aligned [40]. Consequently, in such highly cooperative species it might be better to delegate the decision to an experienced or knowledgeable minority.

## Supplementary Material

Electronic Supplementary Material

## Supplementary Material

Electronic Supplementary Material

## Data accessibility

The dataset supporting this article has been uploaded as part of the electronic supplementary material.
