# Supplementary material for: The influence of the few: a stable ‘oligarchy’ controls information flow in house-hunting ants
Source: Proc Biol Sci. 2018 Feb 14;285(1872):20172726. doi: 10.1098/rspb.2017.2726 (PMC5829206; doi:10.1098/rspb.2017.2726)
Supplement: Electronic Supplementary Material [file rspb20172726supp1.pdf]

The influence of the few: a stable oligarchy controls information flow in house-hunting ants.

Supplementary Information

Thomas O. Richardson[1,2], Charles Mullon[1], James A.R. Marshall[3], Nigel R. Franks[2], and Thomas Schlegel[2]

[1]*Department of Ecology and Evolution, University of Lausanne, Lausanne, Switzerland*
[2]*School of Biological Sciences, University of Bristol, UK*
[3]*Department of Computer Science and Kroto Research Institute, University of Sheffield, UK*

Quantifying tandem run quality

As individual ants were marked with RFID (Radio-Frequency Identification) tags (Fig. S1a), the identities of the leader and follower making up each tandem pair could be recorded. In addition to the participants' identities, the high-resolution overhead video camera was used to extract the break up location of each tandem run (Fig. S1c-d). Finally, to calculate tandem run quality (Fig. S1e), we calculated the net distance travelled towards the target nest (Fig. S1b). Reverse tandem runs – those in which a knowledgeable ant leads a nestmate encountered at a newly-discovered nest back to the old nest – function to recruit lost scouts into an active role in emigrations (Franks et al., 2009). Therefore, for reverse tandem runs we defined the target nest as the nest from which the tandem set out (Fig. S1d).

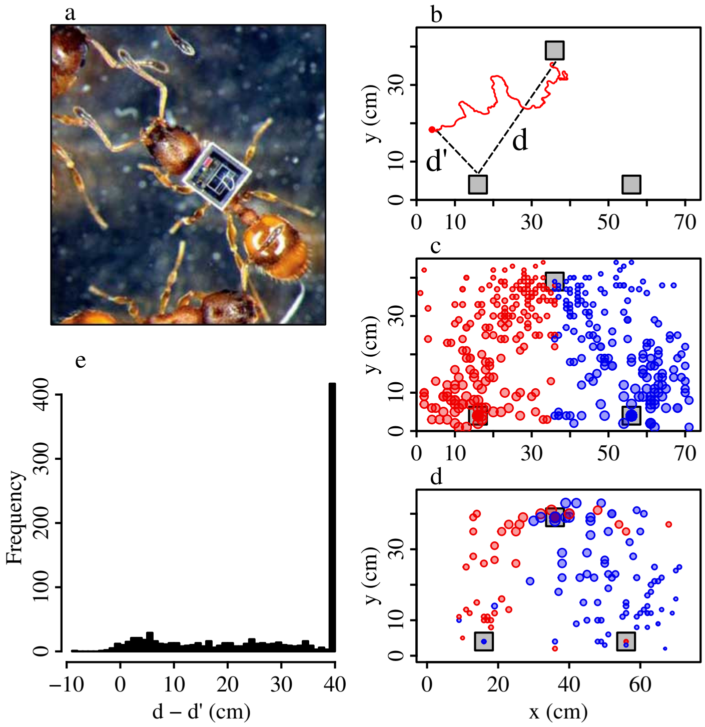

Figure S1: **Defining tandem run quality from the net distance travelled. (a)** *Temnothorax albipennis* worker with a RFID tag glued to the thorax (photo courtesy of N.R. Franks ©). **(b)** Illustration of the calculation of the net distance tracelled for a single tandem run that departed the initial nest (at top), but which broke up before reaching either left or right target nests. The distances d and d' respectively represent the initial and final beeline distance to the target. The, net distance travelled towards the target, is then $d - d'$. **(c)** Spatial distribution of tandem run break-up locations within the arena for (forwards) tandem runs that departed from the original nest. Point colours indicate the identity of the target nest (red; left, blue; right). **(d)** Break-up locations for (reverse) tandem runs. **(e)** Distribution of the net distance travelled towards the target, which is used to calculate tandem quality. Although most tandems reached the target nest, a large proportion broke up before doing so.

Negative degree assortativity

In the main paper we showed that tandem recruitment networks exhibit a negative degree assortativity, that is a negative correlation between the out-degree centrality of an ant and that of its immediate neighbours.

We here calculate the same correlation using *weighted* degree centrality, which is the summed quality of the tandem runs each ant led or followed. We find that individuals that played a major role in the emigration by transmitted a large amount of information by leading tandem runs (high summed out degree quality), tended to be connected to neighbours that transmitted only a little information (Fig. S2a). Conversely, the immediate neighbours of individuals that received a large amount of information (high summed in degree), had neighbours that received little information (Fig. S2b). Hence, as for the regular degree, the weighted degree also exhibits negative degree assortativity: neighbours of ants that were particularly active tandem leaders tended to be particularly inactive leaders, whereas the neighbours of particularly active followers tended to be particularly inactive followers.

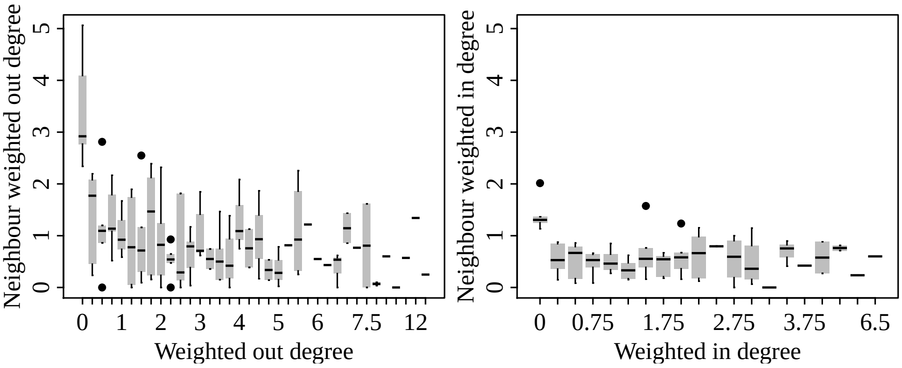

Figure S2: **Negative assortativity for weighted degree centrality. (a)** The summed quality of all tandems an leads is negatively correlated with the summed quality of the tandems led by its nearest neighbours. **(b)** The summed quality of all tandems an follows is negatively correlated with the summed quality of the tandems followed by its nearest neighbours.

Practice predicts performance

In this section we present barplots to confirm the results of the mixed effects models presented in Table 1 in the main paper. The quality of the tandem runs that an ant led showed a positive dependence upon its leading reliability but a negative dependence upon its following reliability, whereas the quality of tandems it followed showed a positive dependece upon its following reliability and no dependence upon its leading reliability The quality of the tandem runs that an ant led showed a positive dependence upon its leading reliability but a negative dependence upon its following reliability (Fig. S3 a,c). Similarly, the quality of tandems an ant followed showed a positive dependece upon its following reliability and no dependence upon its leading reliability (Fig. S3 b,d). Therefore, there is a positive association between the amount of practice within a given role, and the performance in the same role. Furthermore, the negative association between following reliability and the mean quality of tandems led indicates that specialization in one role may inhibit performance in other roles, even when the roles are as closely related as leading and following in tandem runs.

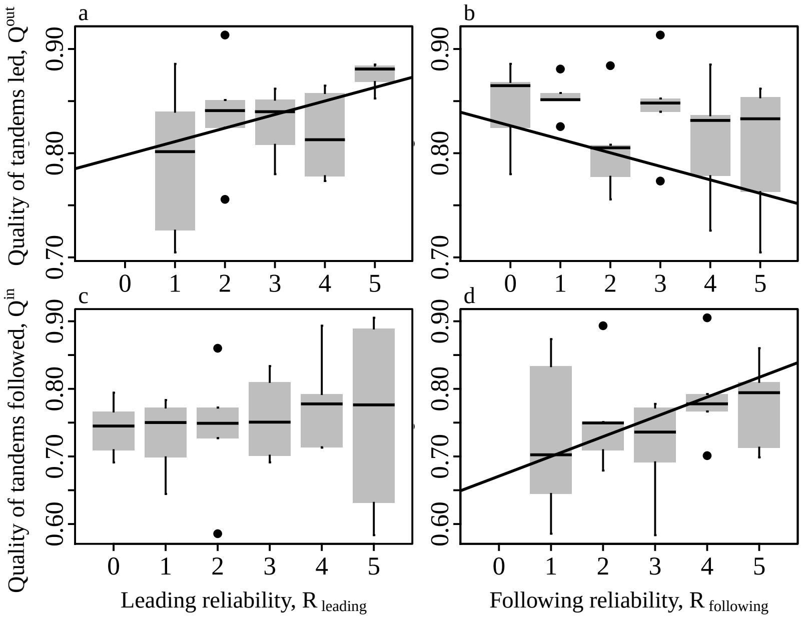

Figure S3: **Individual performance as a leader or follower depends upon practice within the same role.** Evidence that specialization in one role may inhibit performance within the same role: The greater the reliability of an ant in the leading role, the greater the quality of the tandems it led **(a)**, and the greater the reliability of an ant in the following role, the greater the quality of the tandems in which it was a follower **(d)**. **(b)** Evidence that specialization in one role may inhibit performance in other roles: the greater the reliability of an ant in the following role, the *lower* the quality of the tandems it led. **(c)** There was no association between leading reliability and the quality of the tandem runs an individual followed. The fits are given by the linear mixed model presented in Table 1 in the main text.

Testing for assortative matching between leaders and followers

In the main paper, the distribution of leader-follower role reliability differences $f^{\text{tand}}_{\text{Obs}}(\Delta R_{\text{leading}}, \Delta R_{\text{following}})$, was seen to be biased towards positive values. Whilst this demonstrates that tandem runs tend to be composed of a leader that was a more reliable leader than the follower, and a follower that was a more reliable follower than the leader, it does not by itself demonstrate the presence of assortative mathcing.

Therefore, we investigate the statistical significance of this bias. To that end, we compare the observed distribution, $f^{\text{tand}}_{\text{Obs}}(\Delta R_{\text{leading}}, \Delta R_{\text{following}})$, with the equivalent distribution expected in the absence of any association between role reliability and role performance, namely $f^{\text{tand}}_{\text{Exp}}(\Delta R_{\text{leading}}, \Delta R_{\text{following}})$ (Fig. S4a). This expected distribution was generated by constructing randomly-matched tandem runs by repeatedly sampling leaders and followers from the joint role reliability distribution, $f^{ant}(R_{\text{leading}}, R_{\text{following}})$, which is shown in Figure 5a in the main paper.

Plotting the difference between these two distributions, that is, $f^{tand}_{O-E}(\Delta R_{\text{leading}}, \Delta R_{\text{following}})$, confirmed that the observed bias towards positive values of $\Delta R_{\text{leading}}$ and $\Delta R_{\text{following}}$, is real (Fig. S4b), and hence consistent with assortative matching between leaders and followers within tandem pairs.

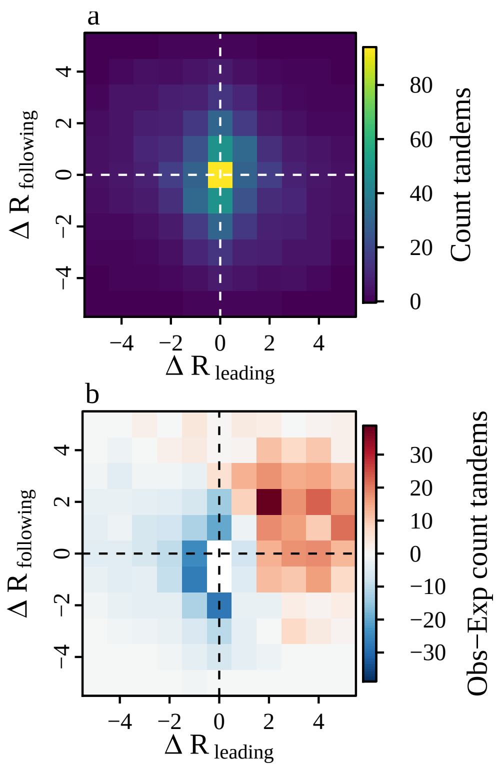

Figure S4: **Differences between the observed and expected leader-follower reliability differences.** **(a)** Reliability differences expected when R_{leading} and $R_{\text{following}}$ are independent, $f^{tand}_{Exp}(\Delta R_{\text{leading}}, \Delta R_{\text{following}})$. **(b)** Signed difference between the observed and expected joint distributions, $f^{tand}_{O-E}(\Delta R_{\text{leading}}, \Delta R_{\text{following}})$. This distribution is skewed towards the upper right quadrat were the tandem leader is a more reliable leader than the tandem follower, and where the follower is a more reliable follower than the leader.

References

Franks, N. R., F.-X. Dechaume-Moncharmont, E. Hanmore, and J. K. Reynolds (2009). Speed versus accuracy in decision-making ants: expediting politics and policy implementation. *Philosophical Transactions of the Royal Society of London B: Biological Sciences 364*(1518), 845–852.
